# MicroRNA Stability in Postmortem FFPE Tissues: Quantitative Analysis Using Autoptic Samples from Acute Myocardial Infarction Patients

**DOI:** 10.1371/journal.pone.0129338

**Published:** 2015-06-05

**Authors:** Yu Kakimoto, Hiroshi Kamiguchi, Eriko Ochiai, Fumiko Satoh, Motoki Osawa

**Affiliations:** 1 Department of Forensic Medicine, Tokai University School of Medicine, Isehara, Kanagawa, Japan; 2 Support Center for Medical Research and Education, Tokai University, Isehara, Kanagawa, Japan; Tokai University, JAPAN

## Abstract

MicroRNAs (miRNAs) are very short (18–24 nucleotides) nucleic acids that are expressed in a number of biological tissues and have been shown to be more resistant to extreme temperatures and pH compared to longer RNA molecules, like mRNAs. As miRNAs contribute to diverse biological process and respond to various kinds of cellular stress, their utility as diagnostic biomarkers and/or therapeutic targets has recently been explored. Here, we have evaluated the usefulness of miRNA quantification during postmortem examination of cardiac tissue from acute myocardial infarction (AMI) patients. Cardiac tissue was collected within one week of the patient’s death and either frozen (19 samples) or fixed in formalin for up to three years (36 samples). RNA integrity was evaluated with an electropherogram, and it appears that longer RNAs are fragmented after death in the long-term fixed samples. Quantitative PCR was also performed for seven miRNAs and three other small RNAs in order to determine the appropriate controls for our postmortem analysis. Our data indicate that miR-191 and miR-26b are more suitable than the other types of small RNA molecules as they are stably detected after death and long-term fixation. Further, we also applied our quantitation method, using these endogenous controls, to evaluate the expression of three previously identified miRNA biomarkers, miR-1, miR-208b, and miR-499a, in formalin-fixed tissues from AMI patients. Although miR-1 and miR-208b decreased (1.4-fold) and increased (1.2-fold), respectively, in the AMI samples compared to the controls, the significance of these changes was limited by our sample size. In contrast, the relative level of miR-499a was significantly decreased in the AMI samples (2.1-fold). This study highlights the stability of miRNAs after death and long-term fixation, validating their use as reliable biomarkers for AMI during postmortem examination.

## Introduction

Sudden death caused by cardiac complications poses significant diagnostic challenges for medical professionals. In fact, ischemic heart failure, the main cause of sudden cardiac death, scarcely displays any abnormal morphological changes 12 h after the initial acute myocardial infarction (AMI) [1]. Histological staining methods, such as triphenyltetrazolium chloride (TTC) staining, are often useful when visualizing the infarcted myocardial area during the sub-acute phase; however, these techniques are incapable of grossly detecting morphological changes in the early phase of AMI [2]. Therefore, it is essential to investigate novel biomarkers whose expression levels change rapidly during AMI. Additionally, in order to be useful during postmortem diagnosis, these biomarkers also need to be detectable in autoptic tissue samples. We previously utilized a proteomic approach using tissues from AMI patients, and revealed that sarcoplasmic sorbin and SH3 domain-containing protein 2 (SORBS2) is released into circulation within 7 h of the initial infarction [3]. Meanwhile, recent reports have demonstrated that some cardiac microRNAs (miRNAs) are significantly elevated in the serum of AMI patients within the first 4 h of infarction, suggesting that changes in the expression of these miRNAs may appear earlier than traditional protein markers [4].

MiRNAs are single-stranded noncoding RNAs comprised of 18 to 24 nucleotides (nt), which can suppress target gene expression at the post-transcriptional level [5]. Notably, miRNAs have been shown to regulate various biological processes in a number of tissues [6, 7], and their stability in blood and urine suggests that they may be the ideal biomarkers for the early diagnosis and prognosis prediction of many diseases. In the last decade alone, a number of studies using human bodily fluids and animal models have revealed promising miRNA biomarkers for various cancers, metabolic disorders, and cardiovascular diseases [8–10]. Moreover, in contrast to vulnerable mRNAs, miRNAs are more resistant to variations in their environments, such as severe changes in pH or temperature as well as repeated ice/thaw cycles [11, 12]. This robustness makes miRNA analysis attractive for investigations conducted postmortem.

Unlike animal tissue sampling in a laboratory, obtaining specimens from human patients is often delayed a significant amount time after death—ranging from several hours if the patient died in a clinical setting/department, to a few days if the patient died outside the hospital. In addition, the tissues obtained during autopsy are usually fixed with formalin, which is necessary for permanent preservation and microscopic analysis, but can severely damage the nucleic acids present in the sample [13]. Indeed, the levels of fragmentation and cross-linking in the DNA and RNA of tissue samples after 24 h of formalin fixation have been shown to reduce both PCR effectiveness [14] and RT-PCR analysis [15]. On the other hand, it was recently demonstrated that embedding the tissues in paraffin after one week of formalin fixation aided miRNA microarray analysis of these samples [16, 17]. However, no systematic study evaluating the influence of postmortem interval or prolonged formalin-fixation on miRNA quantification has been conducted.

In the present study, archival tissue samples were utilized to establish a method to evaluate the miRNA profiles of autoptic tissues following exposure to rough conditions in order to elucidate potential biomarkers of AMI. As cardiac tissues are assumed to be well suited for postmortem quantitative analysis because the heart is less susceptible to degradation than other organs after death [18], we chose to use this tissue in our analysis. We first focused on revealing proper endogenous controls for miRNA quantification by analyzing the stability of 10 small RNAs (smRNAs) in various postmortem intervals and fixation periods. This initial analysis allowed us to then select the appropriate reference RNA controls for our specific protocol and use them to quantify the relative expression levels of specific AMI miRNA biomarkers. Taken together, this study presents a systematic analysis of the effects of postmortem interval and fixation period on miRNA detection in cardiac tissues, highlighting the utility of miRNA quantification in postmortem investigation of sudden cardiac death.

## Materials and Methods

### Tissue sampling and histological analysis

Human cardiac tissue samples were obtained during autopsies performed at the Department of Forensic Medicine, Tokai University School of Medicine between 2011 and 2014. During this time, we collected 19 frozen samples, matched formalin-fixed paraffin-embedded (FFPE) samples, and 17 independent FFPE samples. In order to reduce the effects of individual variability in cardiac damage, we excluded samples obtained from patients that had died fire-related deaths or had severe thoracic trauma. We also omitted ambiguous cases if their time of death had not been determined within ± 2 h. The experimental procedures used in this study were approved by the ethics committee of Tokai University (Med 14I-6). Written informed consent was obtained from the bereaved relatives of all patients to allow experimental use of their tissue samples in this study.

Postmortem interval (PMI) was defined as the time duration from death to sampling. Time of death was confirmed on the basis of witness stories and postmortem phenomena including lividity, rigor mortis, and rectal temperature. PMIs in this study ranged from 10 h to 154 h with an estimated error of ± 2 h. Cadavers were stored at 4°C after a variable period lasting from 3 h to 24 h at room temperature between death and transportation to the morgue.

**Fig 1:**
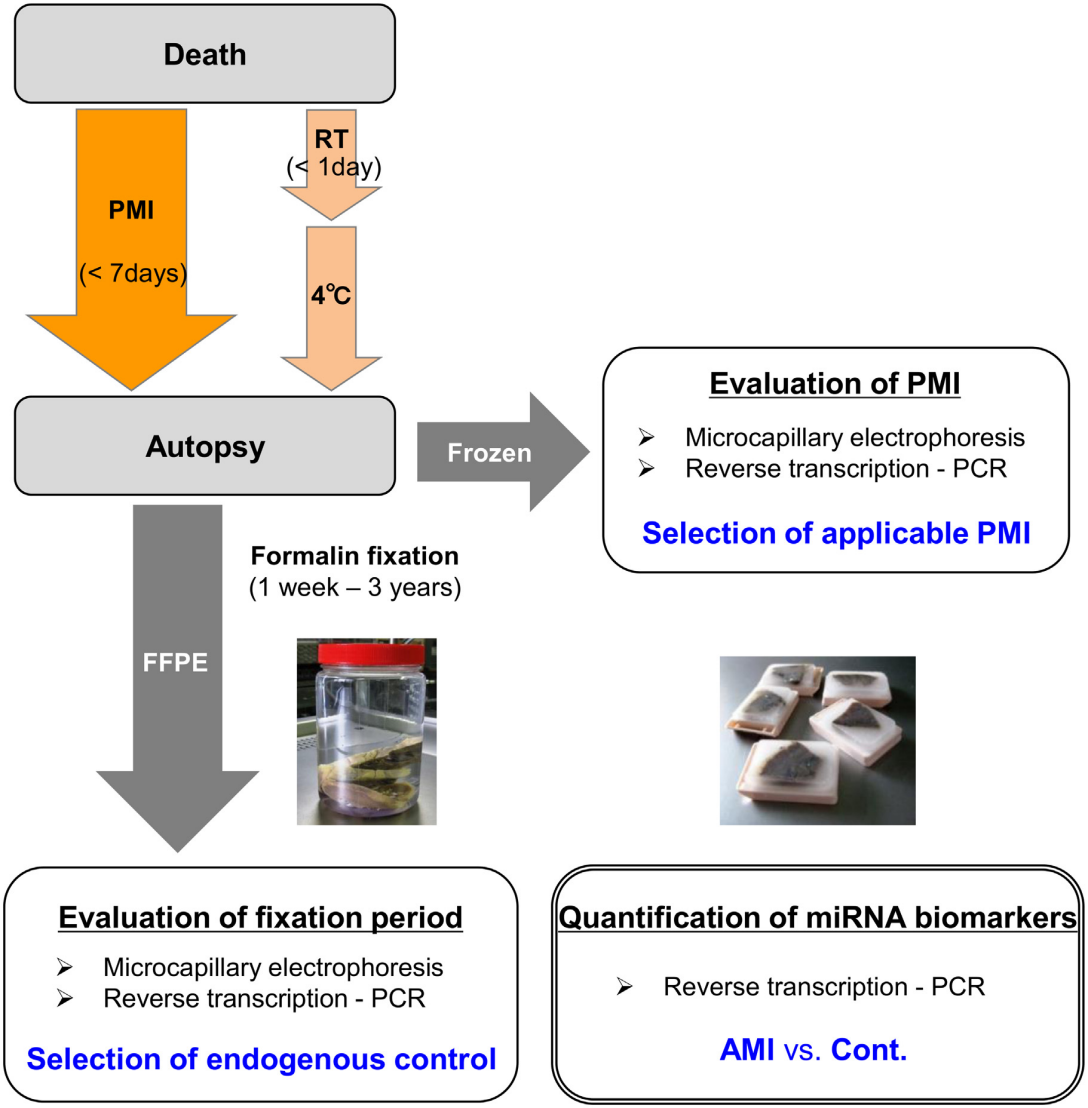
Overall time course of autoptic tissue samples. PMI, postmortem interval; RT, room temperature; FFPE, formalin-fixed paraffin-embedded.

In each autoptic case, ~1-cm-thick transverse sections of the heart (located ~1–3 cm under the atrioventricular sulcus) were removed for sampling. One section was immediately immersed in liquid nitrogen and stored at −80°C. The left free walls of the frozen samples were subjected to RNA isolation within two months. Other heart sections were fixed with 10% formalin, and the fixative solution was renewed twice, one and two days after performing autopsies. Those specimens underwent formalin fixation at room temperature for a duration ranging from 1 week to 37 months. The fixed tissues were then washed with water for 12 h, followed by 6 wash steps with ethanol (70–100%) and 3 wash steps with xylene. The dehydrated tissues were embedded in paraffin at 62°C and stored at room temperature for 1 week to 16 months before analysis. Notably, this length of storage (<2 years) in paraffin has been shown to cause minimal effects on miRNA quantification [16, 17]. The overall time course of sampling is shown in Fig 1.

For histopathological analysis, embedded tissue was sectioned into 4-μm thick slices and stained with hematoxylin and eosin dyes, as well as the Masson trichromal dyes. We examined the left ventricular posterior, lateral, and anterior walls, which are suitable for microscopic observation because of the uniform alignment of myocardial fibers. The septal area was omitted from sampling because its tangled muscular alignment is not appropriate for precise pathological diagnosis, and it also contains Purkinje fibers, which are highly specialized muscles for conduction.

AMI was diagnosed according to the following criteria as previously described [3]: (a) clinical episode of sudden death; (b) increased serum levels of cardiac necrotic proteins, including Troponin T and heart-type fatty acid binding protein; (c) coronary artery occluded by thrombus or severe sclerosis; (d) microscopic changes, including contraction bands and wavy fibers without infiltration of neutrophils. Patients with significant fibrosis and/or fatty metaplasia were excluded in order to focus on the acute phase reaction and to quantify myocardial miRNA accurately. All AMI cases in this study were dead within 12 h of the initial cardiac attack. The causes of death for control cases were hepatic failure, pneumonia, hypothermia, brain injury, blood loss, and drowning. Control hearts showed no macroscopic or microscopic evidence of pathogenesis.

### RNA isolation

RNase inhibitor was sprayed on the workbench before samples were handled, and RNase-free water and equipment were used for miRNA analyses. Total RNA was isolated from frozen tissue samples using an *mir*Vana miRNA Isolation Kit (Applied Biosystems, Foster City, CA) following the manufacture’s protocol. Briefly, approximately 100 mg of crushed tissue was homogenized in 1 ml of Lysis/Binding Buffer. An organic extraction was then performed with 80 μl of miRNA Homogenate Additive and 1 ml of Acid-Phenol-Chloroform. The aqueous phase was added to a filter cartridge and purified RNA was eluted with 100 μl of 95°C Elution Solution.

FFPE tissue samples were cut at a thickness of 20 μm, and one or two sections were used for total RNA isolation with a RecoverAll Total Nucleic Acid Isolation Kit (Applied Biosystems) according to the manufacture’s protocol with the following modifications. The tissue was immersed in 1 mL of Xylene and deparaffinized at 50°C for 5 min. After two washes with ethanol and subsequent centrifugations, the pellet was vacuum dried at room temperature. Protease digestion was performed overnight at 50°C, and then each tube was incubated at 80°C for 15 min. At this stage, the tissue was almost invisible. The lysate was then passed through a filter cartridge and DNase digestion was carried out for 30 min at room temperature. Finally, RNA was eluted with 40 μl of Elution Solution.

RNA concentration and purity were measured for all RNA samples with a spectrophotometer (BioSpec-nano; Shimadzu, Kyoto, Japan). RNA integrity was assessed using microcapillary electrophoresis on a 2100 Bioanalyzer with a Small RNA kit (Agilent Technology). All RNA samples were stored at -80°C until further processing.

### Reverse transcription

For our analysis, we selected 6 candidate reference RNAs that are robustly and consistently expressed in all human tissues, including FFPE specimens [19, 20]. Four candidate biomarkers hypothesized to undergo expression changes during AMI were selected for study [9, 21]. The biomarker candidates are specific to cardiac muscle, or cardiac and skeletal muscle, which are relevant for clinical diagnosis. The length of these RNAs was between 21 and 106 nt (Table 1).

**Table 1:** Control and biomarker candidate specifications.

Gene Symbol	NCBI Accession / miRBase ID	TaqMan ID	Target Sequence	Length(nt)
***Control Candidates***				
U6 snRNA	NR_004394	001973	GTGCTCGCTTCGGCAGCACATATACTAAAATTGGAACGATACAGAGAAGATTAGCATGGCCCCTGCGCAAGGATGACACGCAAATTCGTGAAGCGTTCCATATTTT	106
U47	AF141346	001223	TAATGATTCTGCCAAATGAAATATAATGATATCACTGTAAAACCGTTCCATTTTGATTCTGAGGT	65
RNU6B	NR_002752	001093	CGCAAGGATGACACGCAAATTCGTGAAGCGTTCCATATTTTT	42
miR-191	has-miR-191-5p	002299	CAACGGAAUCCCAAAAGCAGCUG	23
miR-93	hsa-miR-93-3p	002139	ACUGCUGAGCUAGCACUUCCCG	22
miR-26b	has-miR-26b-5p	000407	UUCAAGUAAUUCAGGAUAGGU	21
***Biomarker Candidates***				
miR-1	hsa-miR-1	002222	UGGAAUGUAAAGAAGUAUGUAU	22
miR-133a	hsa-miR-133a-3p	002246	UUUGGUCCCCUUCAACCAGCUG	22
miR-208b	hsa-miR-208b	002290	AUAAGACGAACAAAAGGUUUGU	22
miR-499a	hsa-miR-499a-5p	001352	UUAAGACUUGCAGUGAUGUUU	21

Complementary DNA (cDNA) was synthesized using a TaqMan MicroRNA Reverse Transcription kit (Applied Biosystems) according to the manufacturer’s instructions. Each 15 μl of master mix contained: 5 ng of total RNA, 1× RT primer, 15 nmol of dNTPs, 50 U MultiScribe Reverse Transcriptase, 3.8 U RNase Inhibitor, and 1× RT Buffer. Synthesis was performed in a thermal cycler with a heated lid (Biomerta, Goettingen, Germany) using the following protocol: priming at 16°C for 30 min, transcription at 42°C for 30 min, and enzyme inactivation at 85°C for 5 min. All cDNA samples were stored at -20°C until real-time PCR. Each primer set was also run with no template, and the synthesized product was used as a negative control in the subsequent analyses.

### Real-time PCR

Mature miRNAs were quantified using a StepOnePlus real-time PCR system (Applied Biosystems). Serially diluted cDNAs were used as the template for calculating the PCR efficiency for each primer set. The 10-times diluted cDNAs were also used as the templates in our quantitative analyses. Each 10 μl reaction mixture contained 4.5 μl of cDNA template, 0.5 μl of TaqMan Small RNA Assay Mix, and 5 μl of TaqMan Universal PCR Master Mix II without UNG. The cycling conditions were set as follows: initial polymerase activation at 95°C for 10 min, denaturation at 95°C for 15 sec, and annealing/elongation at 60°C for 1 min with fluorescence acquisition. Data processing was performed using StepOnes software version 2.3 (Applied Biosystems). The threshold value was set at 0.1 throughout this study.

PCR processes were conducted according to the MIQE (minimum information for publication of quantitative real-time PCR) guidelines [22] where applicable. Appropriate reference genes were selected on the basis of Applied Biosystems’ guidelines for the identification of endogenous controls [23].

### Statistical analyses

Electropherograms and real-time PCR data were imported into Microsoft Excel 2010 (Redmond, WA). Linear regression analysis was performed and the coefficient of determination (R^2^) was calculated using the least square method. Standard curves were generated from serially diluted cDNAs for each RNA template using 4 frozen samples (all with PMIs between 0.4 and 6.4 days) and 1 FFPE sample (PMI = 1.3 day, FF = 0.3 month), setting the undiluted samples as 1 arbitrary unit. PCR efficiency (E) was calculated according to the formula: E = 10^-1/slope^—1. Correlation was tested using Pearson’s correlation coefficient, and the Pearson product-moment correlation coefficient (r) was calculated using IBM SPSS ver.19 (Armonk, NY). Triplicate Cq values were averaged, and the relative expression of three specific target miRNAs was determined by the ΔΔCq method [24] using selected endogenous controls. Comparisons between the AMI cases and controls were performed with the Mann-Whitney *U*-test. *P*-values < 0.05 were considered statistically significant in this study.

## Results

### Integrity of small RNA molecules in autoptic tissues

**Fig 2:**
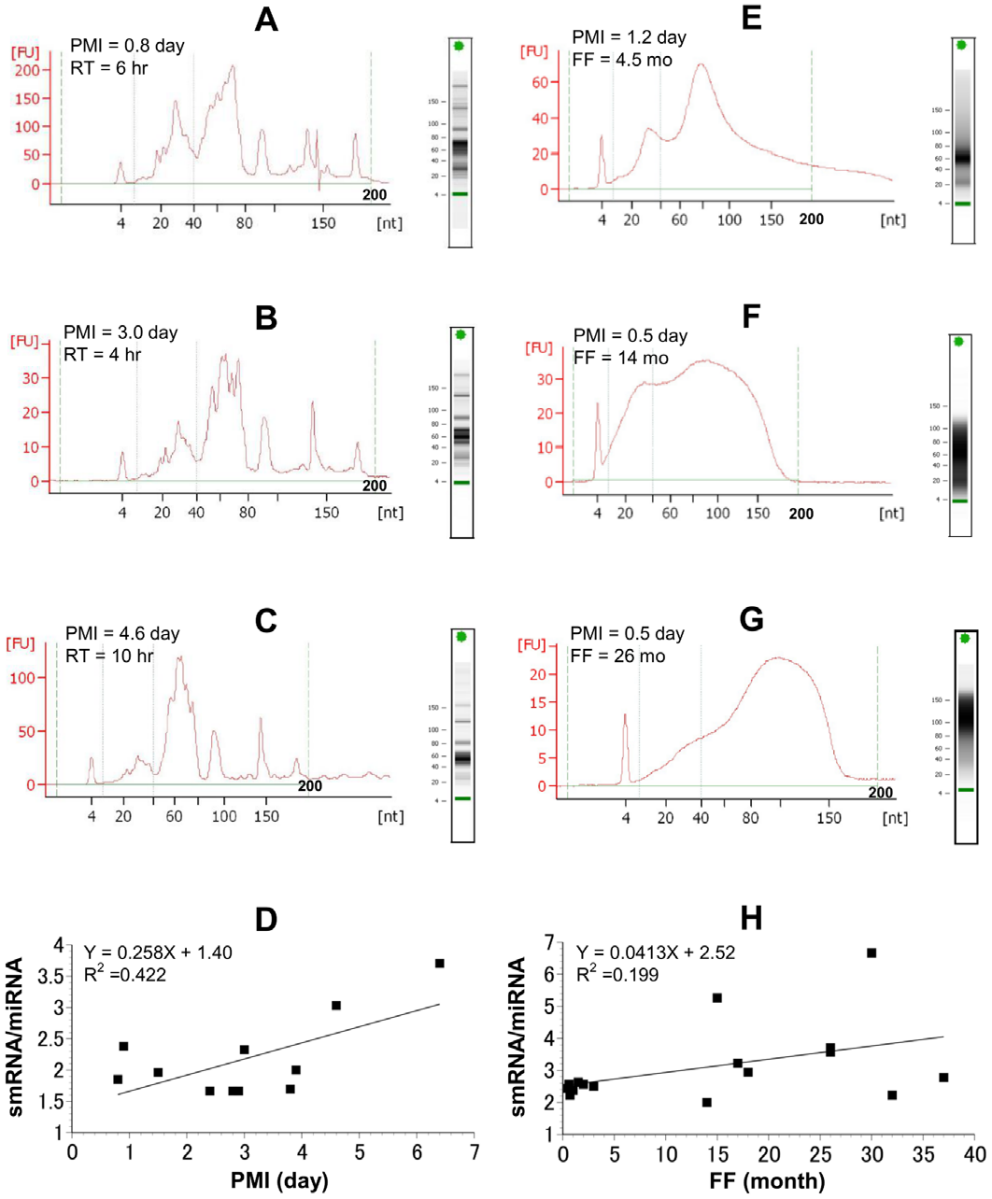
Integrity analysis of smRNAs in autoptic tissues. Representative electropherograms of frozen tissues (A–C) and FFPE tissues (E–G). Green solid lines indicate the area (10–40 nt) containing miRNA peaks, and green dashed lines indicate the area containing smRNA peaks (0–200 nt). The ratio of smRNAs to miRNAs was evaluated using 11 frozen samples (D), and 17 FFPE samples (H). PMI, postmortem interval; RT, room temperature; FF, formalin-fixed period.

In this study, we first sought to evaluate the effects of PMI and fixation on the overall detection profile of smRNA moieties. In the electropherograms obtained from the frozen tissues, we observed multiple peaks characteristic of smRNAs (less than 200 nt, Fig 2A–2C). In fact, the peak between 10 and 40 nt, which has been shown to correspond to miRNAs [25], was clearly visible even a week after death. It is well known that smRNAs show increased long-term storage in tissues and that long RNAs are fragmented during sample degradation. However, the difference in stability between miRNAs (the smallest size of functional RNAs) and other smRNAs in degraded samples has not been fully elucidated. Here we focused on the smRNA/miRNA ratio (concentration of RNAs 0–200 nt in length/concentration of RNAs 10–40 nt in length) in each tissue sample to reveal differences of degradative influences on smRNAs and miRNAs. The smRNA/miRNA ratio increased according to PMI (day) in cases where the cadavers were moved to the morgue within a day of cardiac arrest (r = 0.650, *p* = 0.030; Fig 2D). The miRNA peaks also disappeared and the smRNA/miRNA ratio increased more promptly if the tissues were exposed to room temperature for more than 24 h after death (S1 Fig and S1 Table). Thus, in frozen samples, the total amount of smRNA appears to increase more rapidly than the level of miRNA after death.

Electropherograms were also obtained for our FFPE specimens, all of which had a PMI less than 4 days (including less than 1 day at room temperature and less than 3 days at 4°C). The tissues fixed in formalin for less than 5 months showed clear miRNA peaks on the electropherograms (Fig 2E). However, for samples that underwent more than 1 year of fixation, we observed an RNA smear that covered the whole smRNA area on the electropherogram (Fig 2F and 2G). Compared to the frozen samples, we did not observe a significant increase in the smRNA/miRNA ratio in these FFPE samples (r = 0.444, *p* = 0.074; Fig 2H, S2 Fig, and S2 Table).

### Candidate gene evaluation

#### Amplification efficiency

In order to determine the proper control genes to use in our method, it was essential to first determine if the candidate genes could be efficiently amplified at a high level. PCR efficiency and dynamic range for each candidate are summarized in Table 2 and S3 Table. Due to their grossly outlying amplification efficiency (more than 10%), miR-93 and miR-133a were excluded from further analysis.

**Table 2:** Amplification efficiency of target genes.

Gene Symbol	PCR Efficiency (Mean ± SD)	Dynamic Range*
***Control Candidates***		
U6 snRNA	0.990 ± 0.008	23.81–31.60
U47	0.992 ± 0.015	25.58–33.14
RNU6B	1.027 ± 0.061	27.52–36.03
miR-191	0.999 ± 0.017	25.35–35.30
miR-93	0.823 ± 0.242	30.19–42.84
miR-26b	1.005 ± 0.005	25.06–34.77
***Biomarker Candidates***		
miR-1	1.033 ± 0.038	19.49–30.33
miR-133a	1.123 ± 0.099	18.69–27.73
miR-208b	1.007 ± 0.013	27.19–32.74
miR-499a	0.996 ± 0.009	21.13–31.09

*Dynamic range represents the range of Cq values between the highest and the lowest concentration of generated standard curves.

#### Effect of PMI on candidate gene detection in frozen tissues

**Fig 3:**
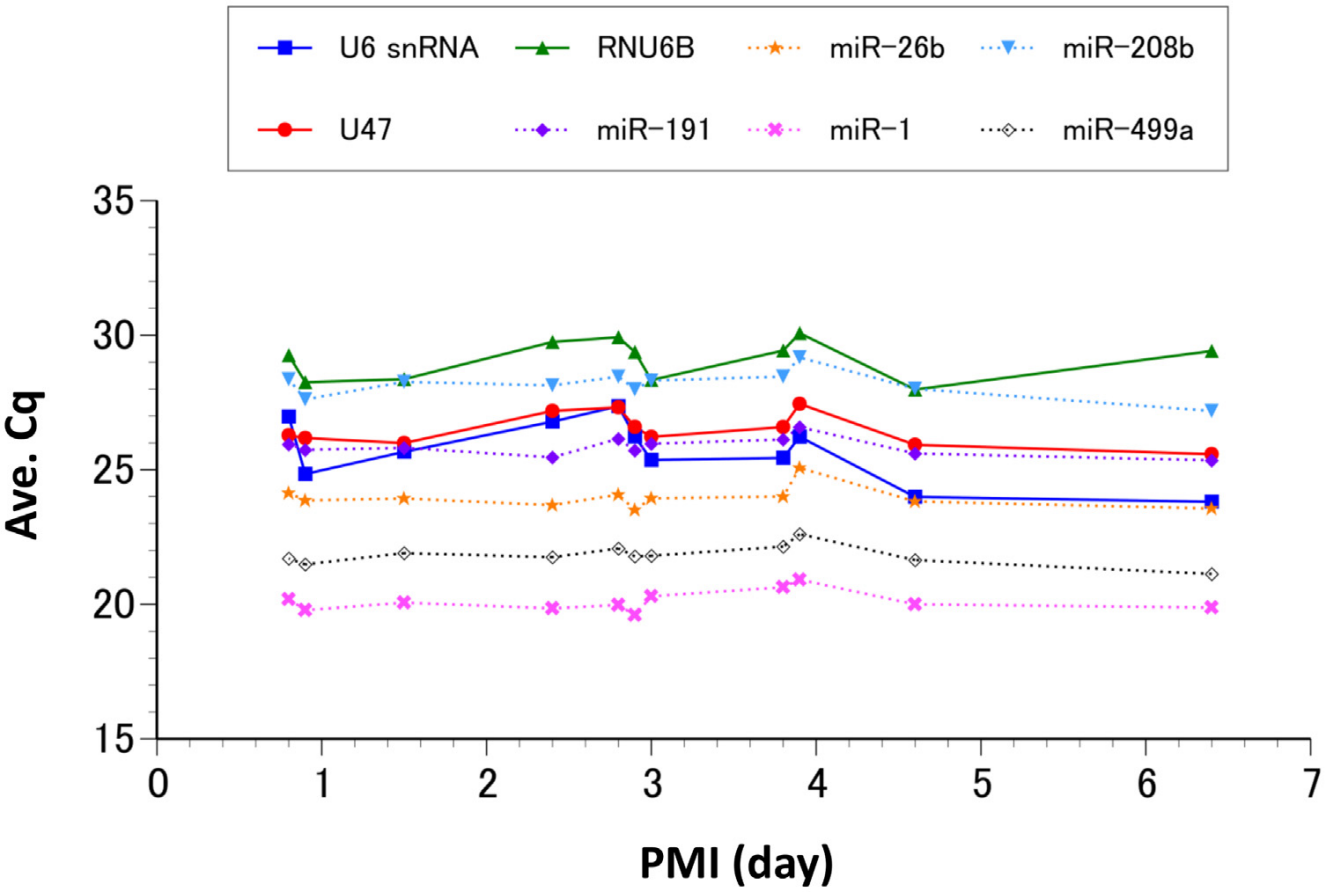
Detection of smRNAs in frozen tissues. Average Cq values (Y-axis) of 8 smRNAs with various postmortem intervals (X-axis) are shown. Cq values for the miRNAs (dashed lines) appear to be more stable than those of other classes of smRNAs (solid lines). The mean value and standard deviation for each candidate smRNA are shown in Table 3.

An endogenous control should be highly and consistently expressed in all tissues studied. Therefore, we analyzed each of our candidate controls along with several previously identified miRNA biomarkers in order to evaluate their stability over time and tissue condition. The amount of each candidate smRNA present in postmortem cardiac tissues did not appear to significantly change in the first week after death if the body was stored at 4°C (Fig 3 and Table 3). However, the standard deviations of the Cq values for the other classes of smRNAs (U6 snRNA, U47, and RNU6B) varied more postmortem than those of the miRNAs analyzed (miR-191, miR-26b, miR-1, miR-208b, and miR-499a).

**Table 3:** Candidate smRNAs in frozen tissues with PMIs up to one week.

Gene Symbol	Cq (Mean ± SD)
*Control Candidates*	
U6 snRNA	25.70 ± 1.16
U47	26.48 ± 0.61
RNU6B	29.11 ± 0.74
miR-191	25.86 ± 0.35
miR-26b	23.96 ± 0.42
*Biomarker Candidates*	
miR-1	20.11 ± 0.39
miR-208b	28.18 ± 0.51
miR-499a	21.82 ± 0.38

Cq, quantification cycle; SD, standard deviation.

#### Effect of formalin-fixation period on candidate gene detection

**Fig 4:**
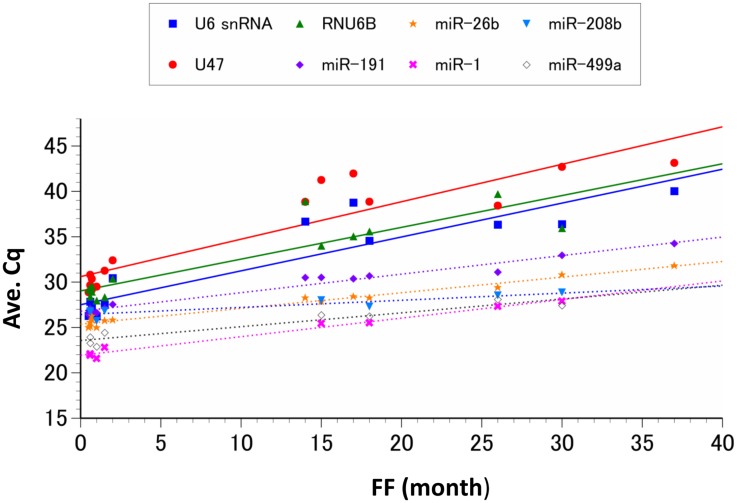
Detection of smRNAs in FFPE tissues. Average Cq values (Y-axis) of 8 smRNAs with various formalin-fixation periods (FF; X-axis) are shown. The detection of every smRNA candidate was reduced as the duration of fixation increased. The rates of degradation for each miRNA (dashed lines) were less than those observed for the other classes of smRNAs (solid lines) analyzed. The regression formula for each smRNA is shown in Table 4.

The Cq values indicated that smRNA detection in the FFPE tissue samples appeared to be closely correlated with the formalin-fixation time (Fig 4 and Table 4). Detection of the larger smRNAs (U6 snRNA, U47, and RNU6B) was also greatly reduced in proportion to the fixation periods. Although the abundance of miRNAs (miR-191, miR-26b, miR-1, miR-208b, and miR-499a) slightly decreased as the fixation period was extended, the rate of this decrease was lower than that of the larger smRNAs. The miRNA that appeared to change the least as fixation time increased was miR-208b, and prior to formalin fixation the Cq values for this miRNA were 2 to 8 cycles higher than the other miRNAs analyzed here. Meanwhile, the miRNAs that were initially detected at a higher level, miR-26b, miR-1, and miR-499a, all decreased to levels lower than that of miR-208b, with miR-26b decreasing below it for samples fixed over 11 months, miR-1 decreasing below it for samples fixed over 36 months, and miR-499a decreasing below it for samples fixed over 39 months.

**Table 4:** Rates of smRNA degradation in FFPE tissues with prolonged fixation up to three years.

Gene Symbol	Regression Formula	R^2^	Degradation Rate (cycle/month)
***Control Candidates***			
U6 snRNA	Y = 0.373X + 27.512	0.832	0.373
U47	Y = 0.413X + 30.608	0.835	0.413
RNU6B	Y = 0.350X + 29.034	0.799	0.350
miR-191	Y = 0.204X + 26.796	0.969	0.204
miR-26b	Y = 0.172X + 25.386	0.971	0.172
***Biomarker Candidates***			
miR-1	Y = 0.205X + 21.944	0.980	0.205
miR-208b	Y = 0.079X + 26.428	0.834	0.079
miR-499a	Y = 0.152X + 23.566	0.910	0.152

Therefore, our data indicate that the detection of the miRNA control genes miR-191 and miR-26b is less influenced by the PMI or fixation time of the sample compared to the other control gene candidates. Both genes also amplified efficiently using PCR. Thus, we chose to utilize miR-191 and miR-26b as our endogenous control genes to quantify the expression of postmortem miRNA biomarkers in deceased cardiac infarction patients. We also chose to exclude over-fixed samples, which were formalin-fixed for more than three months, from further biomarker quantification as the longer fixation periods appeared to increase the degradation variance of each candidate smRNA.

### Quantification of miRNA biomarkers during cardiac infarction

**Fig 5:**
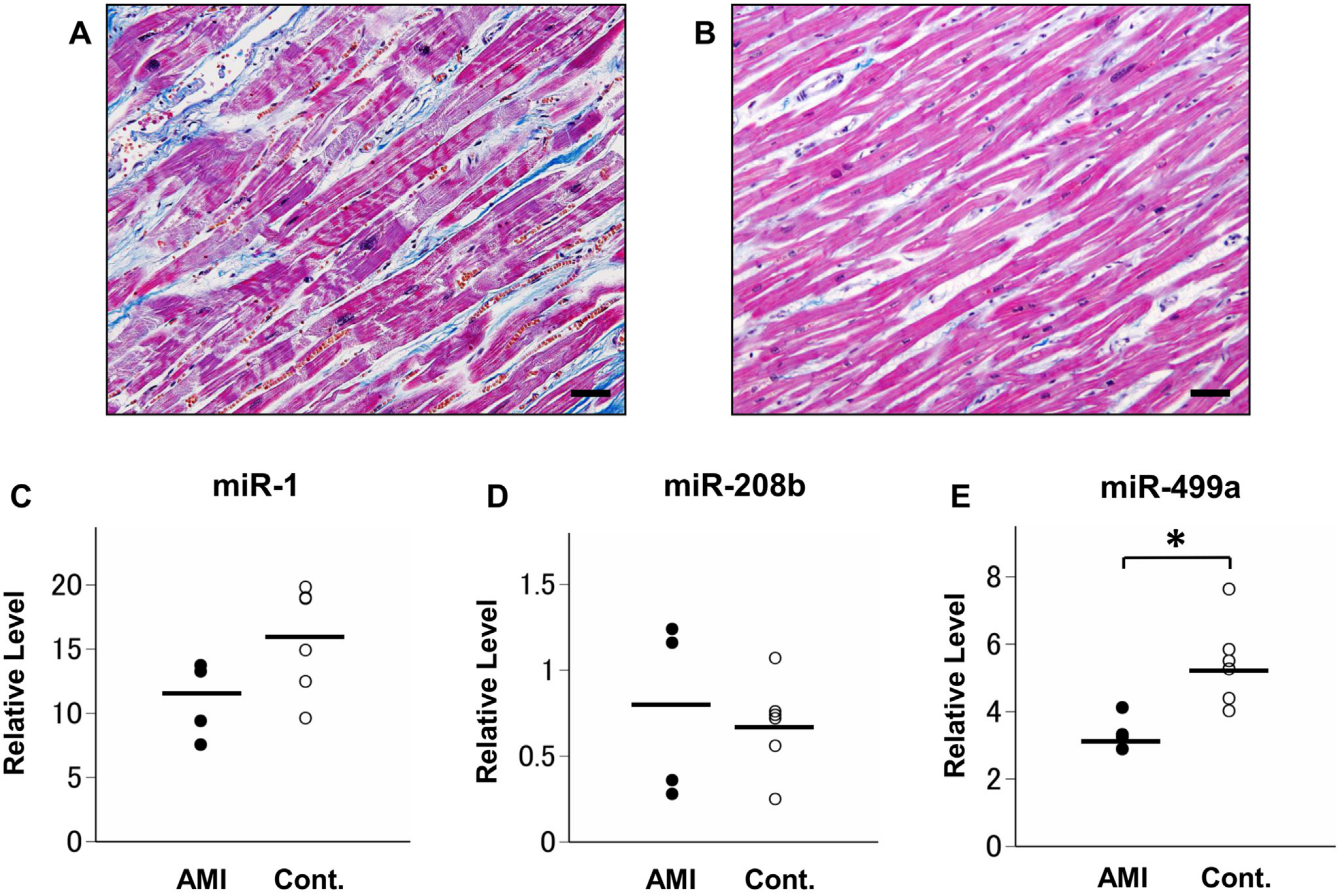
Quantified miRNA biomarker expression in postmortem AMI and control tissues. Histopathological images of human cardiac tissue stained with Masson trichrome highlighting the dominant contraction band necrosis and low levels of neutrophil infiltration in the early phase of AMI (A) and the regular alignment of muscular fibers in the control tissue (B). Scale bar = 50 μm. Relative expression of miR-1 (C), miR-208b (D), and miR-499a (E) in four AMI and seven control cases after normalization to miR-26b and miR-191. *P < 0.05.

The expression levels of the three previously identified miRNA biomarkers, miR-1, miR-208b, and miR-499a, were quantified in four AMI and seven control cases, all of which were formalin-fixed less than two months. The age of the AMI patients and control cases were 62.5 ± 16.2 years and 59.6 ± 15.7 years, respectively. All AMI cases were male and the control group included two female cases. Compared to the control cases, the relative expression of miR-1, miR-208b, and miR-499a in the AMI samples was 0.73-fold (a 1.4-fold decrease; *p* = 0.257), 1.2-fold (*p* = 0.527), and 0.48-fold (a 2.1-fold decrease; *p* = 0.019), respectively (Fig 5).

## Discussion

### Limited evaluation of miRNA integrity

Electrophoretic methods are generally recommended and extensively used to evaluate nucleic acid integrity before further analyses are conducted [26–28]. Furthermore, electropherograms allow the visualization of various sizes of RNAs more clearly than classical gel electrophoresis [29, 30], and have also been shown to be applicable to smRNA [25, 31]. In this study, the electropherograms of tissue samples that had been frozen displayed differentiated miRNA-specific peaks, the size of which noticeably decreased in a PMI-dependent manner after death. In contrast, FFPE samples that underwent prolonged fixation did not have clearly defined miRNA peaks, but instead displayed an RNA smear that was dependent on PMI and fixation time. We believe that during extended fixation longer RNA molecules are fragmented and the resulting RNA particles are also detected in the smRNA area on the electropherogram. This degraded RNA also appeared to interfere with the smRNA concentration measured on the 2100 Bioanalyzer; however, the RT-qPCR analysis using specific primers indicated constant amounts of smRNAs in the frozen samples. Importantly, our electrophoretic analysis of the smRNA profile in archival cardiac tissue is corroborated by previous studies that utilized artificially degraded RNA samples [32]. While these results clearly highlight the drawbacks of assessing miRNA and smRNA integrity using only electrophoretic means, electropherograms are useful in terms of evaluating total RNA concentration (including fragmented and degraded samples). Moreover, the data obtained from frozen postmortem samples suggest that the quantification of smRNAs may be useful for estimating PMI or environmental temperature after death. This insight would be particularly valuable during postmortem investigations when the details of the patient’s death are unclear or ambiguous.

### Selection of endogenous controls

Many previous reports recommend other classes of small noncoding RNAs as endogenous controls for miRNA quantification [19, 33]. It is also well known that such small nuclear RNAs and small nucleolar RNAs are abundantly and consistently expressed across a variety of tissues and cell lines. However, the size of the RNA molecules in these other classes of smRNAs is larger than that of miRNAs, and the effect of this difference in length on quantification, particularly when the sample conditions may result in RNA fragmentation, have not been fully evaluated. Our data indicate that each candidate smRNA was preserved in our tissue samples for at least one week after death. However, there did appear to be more variation in the expression of the larger smRNAs, suggesting that the miRNA candidate genes are less susceptible to environmental change in frozen tissues compared to the other longer types of smRNAs. This difference in stability between the miRNAs and the other classes of smRNAs was even more prominent in the FFPE samples. Although the decrease in detection for all of the smRNAs was highly correlated with the fixation period, each declined at a different rate that appears to be largely dependent on the length of the nucleotide. Thus, the longer RNAs denatured more rapidly than the miRNAs, and only the miRNA controls were detectable within the normal cycling range (Cq < 35) after three years of fixation. It is, therefore, likely that when other classes of smRNAs are used as the endogenous controls for miRNA quantification, the relative miRNA expression levels are overestimated, particularly when the formalin-fixation period is long, regardless of their absolute amounts. These results demonstrate the importance of proper control selection for miRNA quantitative analyses. Our analyses also indicate that in order to avoid the possible negative consequences of long-term tissue fixation, miRNA quantification should ideally be conducted within three months of the formalin fixation. Taken together, the duration of fixation and rate of degradation for each smRNA should be taken into account when quantifying miRNA in preserved FFPE samples.

### Method application: quantification of miRNAs in AMI tissues

After analyzing and validating various potential control molecules, we applied our quantification method to determine the relative expression levels of three previously identified AMI biomarkers, miR-499a, miR-1, and miR-208b, in postmortem AMI and healthy control tissues. MiR-499a has been recognized as a promising AMI biomarker because of its highly specific expression in cardiac muscle. The significant decrease (2.1-fold) in the expression of miR-499a that we observed in the AMI samples in this study is supported by previous reports that miR-499 is highly elevated in the serum of AMI patients (indicative of release from the cardiac tissue) 4 to 12 h after AMI [34, 35] and is greatly decreased in the cardiac infarcted zone in a mouse model of AMI [36].

Similarly, miR-1 is also considered a suitable biomarker of AMI because of its specificity in cardiac muscle as well as skeletal muscle. MiR-1 has also been shown to be remarkably increased in blood samples from patients 4 h after AMI [37, 38] and is reduced in the infarcted myocardia in the mouse model of AMI [39]. Our data show a 1.4-fold decrease in the expression of miR-1 in AMI tissues compared to healthy controls, but this change was not statistically significant. It is possible that the abundant expression of miR-1 in the heart, accounting for 40% of all cardiac miRNA [40], may obscure minute changes in this miRNA during the early phase of AMI, making it difficult to detect significant alterations in expression using our quantitation method.

The third miRNA analyzed, miR-208b, is a particularly interesting AMI biomarker because its expression is not only elevated in the patient’s serum [34, 41], but has also been shown to increase in cardiac tissue during AMI [42]. Interestingly, while miR-208b is known to play an important role during fetal cardiac development, it is scarcely expressed in the adult heart except during cardiac stress, such as ischemia and hypertrophy [43, 44]. Our data show a slight elevation (1.2-fold) in the expression of miR-208b in AMI tissues, but lacked statistical significance. We suspect that because of its low abundance in the adult heart, the expression of miR-208b may be more affected by PMI and individual patient background than other miRNAs.

### Limitations

First, it is likely that the small sample size lowered the levels of statistical significance for miRNA biomarker candidates. However, we selected AMI cases with the following strict criteria; sudden death episode, blood tests, severe coronary obstruction, and microscopic changes, and omitted cases where any of the four criteria were not matched. Therefore, the number of patients was limited in this study.

Second, the heterogeneous causes of death in control cases may have affected miRNA expression. Certain kinds of cardiac injury occur in severely ill patients that differ from acute coronary syndromes. For example, subendocardial haemorrhages are often observed after blood loss or brain injury [45]. As most of the severe cardiac injuries accompany histopathological changes, we excluded such cases from the control cases in this study. However, the expression of some cardiac miRNAs being altered prior to microscopic changes occurring remains a possibility. Although heterogeneity is inevitable in the forensic field, where researchers often have to distinguish the primary cardiac death from other secondary cardiac failure, the variety of control cases may also limit the statistical significance in this case control study.

However, it should be noted that the overall changes/trends in the expression of each miRNA biomarker in the archival tissues used in this study do in fact confirm the findings of previous studies that used human serum and animal models. Therefore, additional high throughput analyses with a larger number of patients are necessary to strengthen the practical utility of miRNA at postmortem investigation using autoptic tissue samples.

## Conclusions

In the present study, we have systematically analyzed various controls and biomarkers for their use in miRNA quantification in addition to determining the effects of PMI and tissue treatment on miRNA detection in postmortem cardiac samples. Our results indicate that it is essential to consider the duration of fixation and PMI during the selection of appropriate endogenous controls. Furthermore, the miRNAs analyzed here appear to be more resistant to rough treatment conditions compared to other smRNAs, leading us to utilize miR-191 and miR-26b as endogenous controls in our quantitative analysis. Although our sample size and heterogeneous control cases lowered the significance of some miRNA biomarkers in this study, miR-499a levels were significantly decreased in AMI cardiac tissues. We believe our method of miRNA quantification is practically useful at postmortem examination, and can be a helpful diagnostic tool for critical cardiac injury. Future studies will fully assess the possibility of postmortem miRNA analysis, to clarify the various causes of sudden death.

## Supporting Information

S1 Fig Electropherograms of frozen tissue samples. The solid green lines indicate the area containing miRNA peaks (10–40 nt), and the green dashed lines indicate the area containing smRNA peaks (0–200 nt). Details of each sample are shown S1 Table.
(PPTX)

S2 Fig Electropherograms of FFPE tissue samples. The solid green lines indicate the area containing miRNA peaks (10–40 nt), and the green dashed lines indicate the area containing smRNA peaks (0–200 nt). Details of each sample are shown in S2 Table.
(PPTX)

S1 Table Individual data for the samples shown in S1 Fig. (DOCX)

S2 Table Individual data for the samples shown in S2 Fig. (DOCX)

S3 Table Standard curves for 10 smRNA primers. (DOCX)
