# Supplementary figures and images for: MicroRNA Stability in Postmortem FFPE Tissues: Quantitative Analysis Using Autoptic Samples from Acute Myocardial Infarction Patients
Source: PLoS One. 2015 Jun 5;10(6):e0129338. doi: 10.1371/journal.pone.0129338 (PMC4457786; doi:10.1371/journal.pone.0129338)

## Slide 1
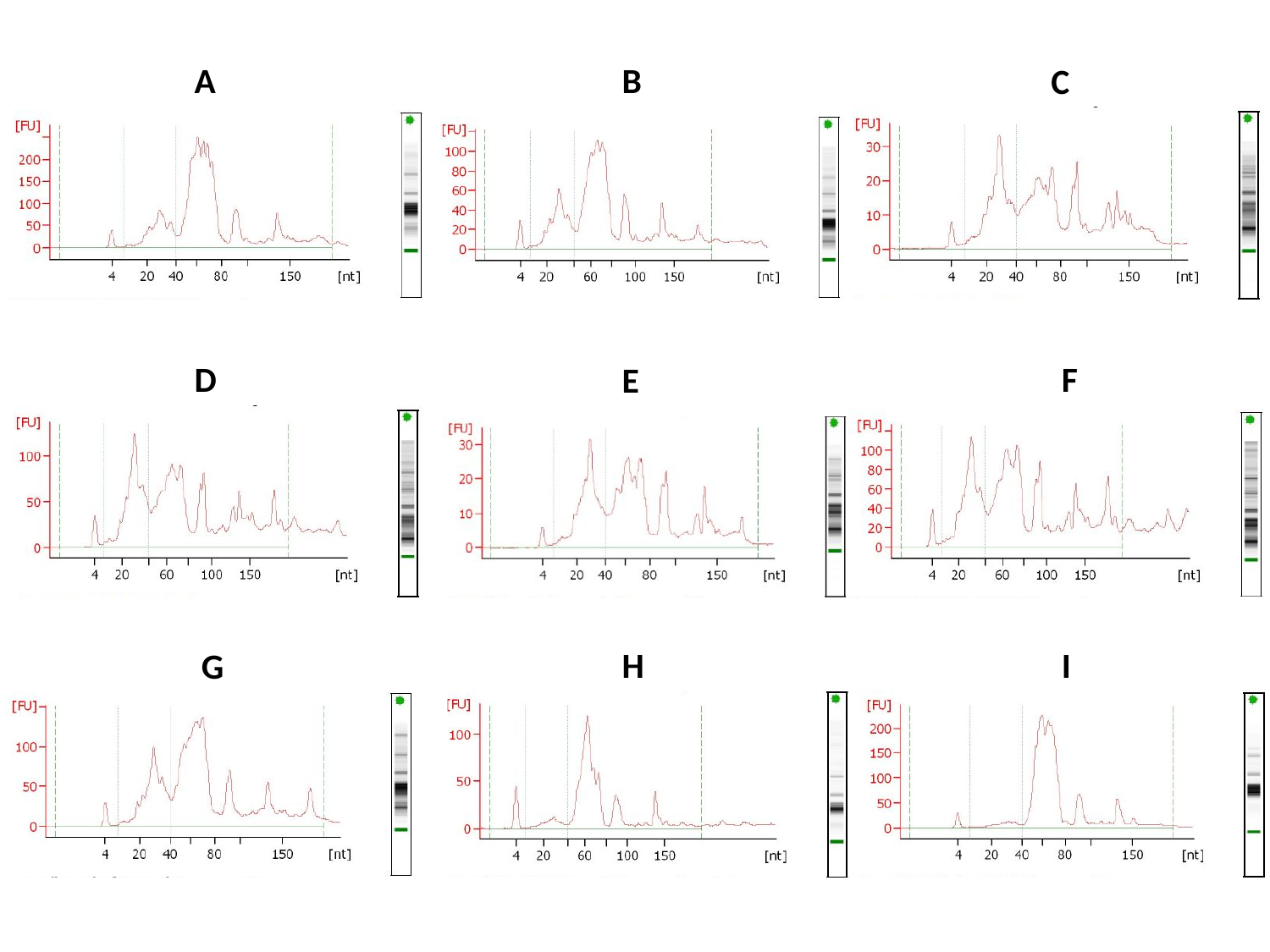

A
B
C
D
F
E
H
I
G

Supplement: S1 Fig — The solid green lines indicate the area containing miRNA peaks (10–40 nt), and the green dashed lines indicate the area containing smRNA peaks (0–200 nt). Details of each sample are shown S1 Table. (PPTX) [file pone.0129338.s001.pptx]

## Slide 1
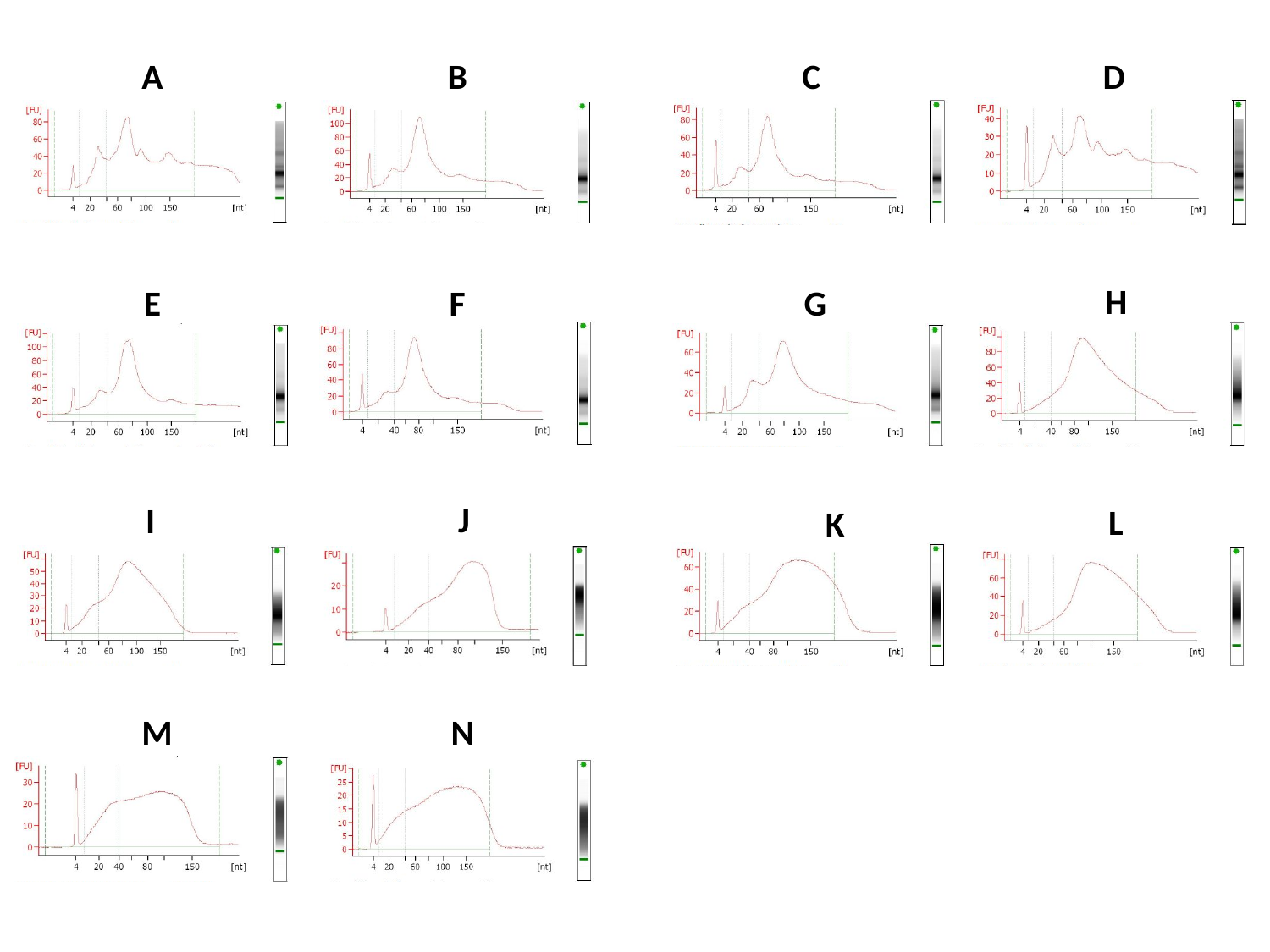

A
B
C
D
H
E
F
G
J
I
L
K
M
N

Supplement: S2 Fig — The solid green lines indicate the area containing miRNA peaks (10–40 nt), and the green dashed lines indicate the area containing smRNA peaks (0–200 nt). Details of each sample are shown in S2 Table. (PPTX) [file pone.0129338.s002.pptx]
