# Supplementary material for: MicroRNA Stability in Postmortem FFPE Tissues: Quantitative Analysis Using Autoptic Samples from Acute Myocardial Infarction Patients
Source: PLoS One. 2015 Jun 5;10(6):e0129338. doi: 10.1371/journal.pone.0129338 (PMC4457786; doi:10.1371/journal.pone.0129338)
Supplement: S1 Table — (DOCX) [file pone.0129338.s003.docx]

|  | **total PMI**  **(day)** | **PMI at RT**  **(hour)** | **smRNA / miRNA** |
| --- | --- | --- | --- |
| **A** | 0.9 | 5 | 2.40 |
| **B** | 1.5 | 3 | 1.94 |
| **C** | 2.4 | 12 | 1.68 |
| **D** | 2.8 | 8 | 1.66 |
| **E** | 2.9 | 8 | 1.68 |
| **F** | 3.8 | 3 | 1.69 |
| **G** | 3.9 | 7 | 1.98 |
| **H** | 6.4 | 7 | 3.71 |
| **I** | 3.5 | 24 | 7.29 |

PMI, postmortem interval; RT, room temperature.
