# Supplementary material for: MicroRNA Stability in Postmortem FFPE Tissues: Quantitative Analysis Using Autoptic Samples from Acute Myocardial Infarction Patients
Source: PLoS One. 2015 Jun 5;10(6):e0129338. doi: 10.1371/journal.pone.0129338 (PMC4457786; doi:10.1371/journal.pone.0129338)
Supplement: S2 Table — (DOCX) [file pone.0129338.s004.docx]

|  | **total PMI**  **(day)** | **PMI at RT**  **(hour)** | **FF**  **(month)** | **smRNA / miRNA** |
| --- | --- | --- | --- | --- |
| **A** | 1.3 | 5 | 0.5 | 2.43 |
| **B** | 0.8 | 3 | 0.6 | 2.52 |
| **C** | 2.0 | 12 | 0.6 | 2.55 |
| **D** | 2.2 | 5 | 0.7 | 2.22 |
| **E** | 2.5 | 12 | 1.0 | 2.41 |
| **F** | 1.4 | 4 | 1.5 | 2.60 |
| **G** | 3.1 | 11 | 2.0 | 2.55 |
| **H** | 1.1 | 8 | 15 | 5.38 |
| **I** | 1.9 | 13 | 17 | 3.20 |
| **J** | 2.1 | 15 | 18 | 2.93 |
| **K** | 0.5 | 3 | 26 | 3.67 |
| **L** | 0.7 | 9 | 30 | 6.81 |
| **M** | 1.0 | 5 | 32 | 2.24 |
| **N** | 1.1 | 3 | 37 | 2.75 |

PMI, postmortem interval; RT, room temperature; FF, formalin-fixed period.
