# Supplementary material for: MicroRNA Stability in Postmortem FFPE Tissues: Quantitative Analysis Using Autoptic Samples from Acute Myocardial Infarction Patients
Source: PLoS One. 2015 Jun 5;10(6):e0129338. doi: 10.1371/journal.pone.0129338 (PMC4457786; doi:10.1371/journal.pone.0129338)
Supplement: S3 Table — (DOCX) [file pone.0129338.s005.docx]

**S3 Table.** Standard curves for 10 smRNA primers.

| **Sample ID*** | **Gene Symbol** | **U-6 snRNA** | **U-47** | **RNU6B** | **miR-191** | **miR-93** | **miR-26b** | **miR-1** | **miR-133a** | **miR-208b** | **miR-499a** |
| --- | --- | --- | --- | --- | --- | --- | --- | --- | --- | --- | --- |
| **Frozen 1** | **Slope** | -3.352 | -3.307 | -3.014 | -3.343 | -4.189 | -3.309 | -3.367 | -2.885 | -3.288 | -3.370 |
|  | **Y-Intercept** | 23.269 | 24.002 | 24.472 | 23.047 | 37.820 | 21.823 | 16.160 | 17.570 | 25.372 | 18.830 |
|  | **R^2^** | 0.998 | 0.997 | 0.999 | 1.000 | 0.948 | 0.984 | 1.000 | 0.993 | 1.000 | 0.998 |
|  | **PCR Efficiency** | 0.988 | 1.006 | 1.147 | 0.991 | 0.733 | 1.005 | 0.982 | 1.221 | 1.014 | 0.980 |
|  | **Range** | 26.636-30.632 | 27.276-30.844 | 27.522-31.152 | 26.390-30.414 | 31.938-36.926 | 25.059-28.530 | 19.525-22.256 | 20.416-22.930 | 28.670-31.649 | 22.206-25.290 |
| **Frozen 2** | **Slope** | -3.353 | -3.342 | -3.324 | -3.325 | -3.337 | -3.306 | -3.260 | -2.941 | -3.243 | -3.334 |
|  | **Y-Intercept** | 24.161 | 25.768 | 28.598 | 23.888 | 28.870 | 21.672 | 16.371 | 17.910 | 26.483 | 20.010 |
|  | **R^2^** | 1.000 | 1.000 | 0.992 | 0.993 | 0.958 | 0.997 | 0.945 | 0.996 | 0.996 | 0.996 |
|  | **PCR Efficiency** | 0.987 | 0.992 | 0.999 | 0.999 | 0.994 | 1.007 | 1.027 | 1.188 | 1.034 | 0.995 |
|  | **Range** | 27.515-30.993 | 29.118-33.141 | 32.023-36.025 | 27.062-31.126 | 32.328-35.716 | 24.945-27.880 | 19.494-21.843 | 21.886-23.472 | 29.655-32.620 | 23.269-26.317 |
| **Frozen 3** | **Slope** | -3.329 | -3.323 | -3.326 | -3.322 | -3.327 | -3.327 | -3.335 | -3.255 | -3.305 | -3.314 |
|  | **Y-Intercept** | 22.922 | 24.128 | 26.787 | 23.254 | 28.772 | 21.745 | 17.579 | 16.354 | 25.872 | 19.285 |
|  | **R^2^** | 0.998 | 1.000 | 0.996 | 1.000 | 0.999 | 1.000 | 0.997 | 0.996 | 0.999 | 1.000 |
|  | **PCR Efficiency** | 0.997 | 0.999 | 0.998 | 1.000 | 0.998 | 0.998 | 0.995 | 1.029 | 1.007 | 1.003 |
|  | **Range** | 26.225-29.982 | 27.448-30.725 | 30.077-33.095 | 26.587-30.578 | 32.068-36.074 | 25.070-29.076 | 21.288-23.895 | 19.570-22.471 | 29.450-32.177 | 22.606-25.595 |
| **Frozen 4** | **Slope** | -3.320 | -3.322 | -3.382 | -3.353 | -3.312 | -3.328 | -3.160 | -3.326 | -3.355 | -3.331 |
|  | **Y-Intercept** | 20.492 | 22.304 | 25.887 | 21.970 | 26.914 | 20.241 | 16.720 | 15.429 | 23.691 | 17.840 |
|  | **R^2^** | 1.000 | 0.999 | 0.992 | 1.000 | 0.997 | 0.993 | 1.000 | 0.986 | 0.985 | 0.994 |
|  | **PCR Efficiency** | 1.001 | 1.000 | 0.976 | 0.987 | 1.004 | 0.997 | 1.072 | 0.998 | 0.986 | 0.996 |
|  | **Range** | 23.809-27.443 | 25.579-29.603 | 29.415-33.449 | 25.349-29.387 | 30.192-35.538 | 23.559-27.636 | 27.240-22.737 | 18.686-21.276 | 27.185-30.212 | 21.125-23.510 |
| **FFPE 1** | **Slope** | -3.373 | -3.412 | -3.286 | -3.228 | -7.084 | -3.319 | -3.255 | -3.277 | -3.319 | -3.335 |
|  | **Y-Intercept** | 24.130 | 25.280 | 25.357 | 25.768 | 43.430 | 24.131 | 19.496 | 19.366 | 26.386 | 22.968 |
|  | **R^2^** | 0.998 | 0.980 | 1.000 | 0.996 | 0.739 | 0.999 | 0.998 | 0.999 | 0.999 | 0.992 |
|  | **PCR Efficiency** | 0.979 | 0.964 | 1.015 | 1.041 | 0.384 | 1.001 | 1.029 | 1.019 | 1.001 | 0.995 |
|  | **Range** | 27.582-31.604 | 28.467-32.620 | 28.640-32.597 | 28.958-32.442 | 34.660-42.842 | 27.470-30.951 | 22.725-25.639 | 22.622-25.273 | 29.741-32.737 | 26.248-29.640 |

Frozen 1, PMI = 0.4 day (RT 5h); Frozen 2, PMI = 1.3 day (RT 14h); Frozen 3: PMI = 3.9 day (RT 7h); Frozen 4: PMI = 6.4 day (RT 7h); FFPE 1, PMI = 1.3 day, (RT 4h) and FF = 0.3 month.
